# MiR-152 May Silence Translation of CaMK II and Induce Spontaneous Immune Tolerance in Mouse Liver Transplantation

**DOI:** 10.1371/journal.pone.0105096

**Published:** 2014-08-18

**Authors:** Yan Wang, Yang Tian, Yuan Ding, Jingcheng Wang, Sheng Yan, Lin Zhou, Haiyang Xie, Hui Chen, Hui Li, Jinhua Zhang, Jiacong Zhao, Shusen Zheng

**Affiliations:** 1 Key Laboratory of Combined Multi-organ Transplantation, Ministry of Public Health, First Affiliated Hospital, Zhejiang University School of Medicine, Hangzhou, China; 2 Division of Hepatobiliary and Pancreatic Surgery, First Affiliated Hospital, Zhejiang University School of Medicine, Hangzhou, China; The University of Hong Kong, Hong Kong

## Abstract

Spontaneous immune tolerance in mouse liver transplantation has always been a hotspot in transplantation-immune research. Recent studies revealed that regulatory T cells (Tregs), hepatic satellite cells and Kupffer cells play a potential role in spontaneous immune tolerance, however the precise mechanism of spontaneous immune tolerance is still undefined. By using Microarray Chips, we investigated different immune regulatory factors to decipher critical mechanisms of spontaneous tolerance after mouse liver transplantation. Allogeneic (C57BL/6-C3H) and syngeneic (C3H-C3H) liver transplantation were performed by 6-8 weeks old male C57BL/6 and C3H mice. Graft samples (N = 4 each group) were collected from 8 weeks post-operation mice. 11 differentially expressed miRNAs in allogeneic grafts (Allografts) vs. syngeneic grafts (Syngrafts) were identified using Agilent Mouse miRNA Chips. It was revealed that 185 genes were modified by the 11 miRNAs, furthermore, within the 185 target genes, 11 of them were tightly correlated with immune regulation after Gene Ontology (GO), Kyoto Encyclopedia of Genes and Genomes (KEGG) analysis and Genbank data cross-comparison. Verified by real-time PCR and western blot, our results indicated that mRNA expression levels of IL-6 and TAB2 were respectively down regulated following miR-142-3p and miR-155 augment. In addition, increased miR-152 just silenced mRNA of CaMK II and down-regulated translation of CaMK II in tolerated liver grafts, which may play a critical role in immune regulation and spontaneous tolerance induction of mouse liver transplantation.

## Introduction

Liver transplantation is an established therapeutic option for acute and chronic end-stage liver diseases, metabolic diseases and early hepatocellular carcinoma [1]. However, donor shortage and side effects of immunosuppressants are the two major issues that hamper the progress of liver transplantation. Donor shortage has forced transplant teams to explore new methods to increase the potential donor pool [2]. On the other hand, immunosuppressant is still needed for recipients, meanwhile, the side effects and complications such as infection and tumor recurrence have always been the vexing challenges for clinical physicians [3].

The ability to produce a tolerant state after transplantation would potentially obviate long-term immunosuppressant usage. For decade of years, researches have been done to demonstrate the mechanisms of graft dysfunction and immune tolerance. Qian demonstrated hepatic satellite cells have potent immunoregulatory activity via B7-H1-mediated induction of apoptosis in activated T cells [4]. Ye Y. and colleagues provided new evidence of the potential regulatory effects of galectin-1 in allogeneic immune responses in a murine model of liver transplantation [5]. Tregs control immune responses to foreign and allo-antigens and could induce tolerance [6]. Rapamycin has beneficial effects on Tregs' biology compared with calcineurin inhibitor in potentially attaining host hyporesponsiveness to an allograft [7], [8]. And the concept “clinical operational tolerance” is proposed and used clinically in recent years, and biopsies are designed to study markers of operational tolerance and to monitor for subclinical events [9]–[11]. However, the exact mechanisms involved in the complicated immune system to achieve tolerance remain unclear, and the results of clinical operational tolerance are still unpersuasive.

MicroRNAs (miRNAs), an abundant class of approx 22-nucleotide small RNAs that control gene expression at the posttranscriptional level, may impact lymphocyte development or function and play important roles in transplant immunology. Recent studies revealed that miRNAs might participate in the regulation of the HLA-G gene expression through a putative miRNA binding site at its 3' UTR region [12]. Specific miRNAs could govern expression of genes relevant to allograft rejection, tolerance induction and post-transplant infection [13]. Besides, they were also monitored as biomarkers in organ quality, ischemia-reperfusion injury, acute rejection, tolerance and chronic allograft dysfunction [14].

In murine model, MHC-mismatched liver grafts could be spontaneously accepted and reach immune tolerance without immunosuppressant [5], [15], which provides us an ideal model to investigate the mechanisms of spontaneous immune tolerance. However, the changes of miRNA in tolerated mouse liver graft are still unprofiled.

In this study, we illuminated miRNA changes in mouse tolerant liver transplantation model by using microarray chip and further identified the important spots of miRNAs and their target genes in inducing tolerance of liver graft. Our observations offer novel findings about potential mechanism of spontaneous immune tolerance for clinic application.

## Materials and Methods

### Mice

6–8 weeks old male inbred C57BL/6 (H2^b^) and C3H (H2^k^) mice were purchased from the Animal Research Institution of Zhejiang Province (Hangzhou, China). Mice were housed under a standard SPF environment with a 12h dark-light cycle and free access to water and food. All animal experiments were conducted in accordance with the Guidelines for the Care and Use of Laboratory Animals and were approved by the Animal Ethics Review Committees of Zhejiang University.

### Orthotropic liver transplantation and Sample Collection

C57BL/6 or C3H mice weighing 23∼25g were used as donors and C3H mice weighing 24∼26 g were used as recipients for allogeneic or syngeneic liver transplantation, respectively. Isoflurane was administrated as a general inhalation anesthetic in all case. All surgical processing was performed by two surgeons who have license for animal surgery using a combined cuff and suture technique as described in previous study [16]. The hepatic artery was not reconstructed. The warm ischemia/cold ischemia time was strictly controlled to eliminate the deviation caused by ischemia-reperfusion injury. After the postoperative restoration of temperature and rehydration, mice were sent to individual ventilated cages for housing. 8 weeks post-transplantation, allografts and syngrafts as well as normal liver of C57BL/6, C3H mice (N = 4 for each group) were collected and stored in ultra-low temperature refrigerator.

### Total RNA Isolation and Quality Control

Total RNA of graft samples were extracted and purified using mirVanaTM Isolation Kit (Cat#AM1560,Ambion,Austin,TX,USA) following the manufacturer's instructions and checked for a RIN number to inspect RNA integration by an Agilent Bioanalyzer 2100 (Santa Clara,CA,USA).

### Microarray Chip Analysis

Isolated RNA was analyzed on Agilent mouse miRNA (8*15K; ID: 21828) V12.0 Chips. Briefly, miRNA molecular in total RNA was labeled and hybridized with 100 ng Cy3-labeled RNA by miRNA Complete Labeling and Hyb Kit (Santa Clara, CA, USA) in hybridization Oven (Santa Clara, CA, USA) at 55°C, 20rpm for 20 hours. After hybridization, slides were washed in staining dishes (Cat# 121, Thermo Shandon, and Waltham, MA, USA) with Gene Expression Wash Buffer Kit (Cat# 5188-5327, Agilent technologies, Santa Clara, CA, US). Slides were scanned by Agilent Microarray Scanner and Feature Extraction software 10.7 with default settings. Raw data were normalized by Quantile algorithm, Gene Spring Software 11.0 (Agilent technologies, Santa Clara, CA, US).

All the detailed miRNA microarray information was displayed in the public database of SBC Analysis System (http://sas.ebioservice.com/). Data was available through username BH2010658 with password 091912. Differentially expressed miRNAs were defined by fold changes of detected signals.

### GO and KEGG pathway analysis for predicted gene targets of differentially expressed miRNAs

The online software—TargetScanMouse 6.0 was used for microRNA target prediction (http://www.targetscan.org/mmu_61/) in conjunction with miRanda (http://www.microrna.org/microrna)and miRbase (http://www.mirbase.org/). The target genes of differentially expressed miRNA were predicted with total context score >−0.40 and then the targets were analyzed in terms of their GO categories and KEGG pathways, by using the online tool named “Database for Annotation, Visualization and Integrated Discover” (http://david.abcc.ncifcrf.gov/).

### Quantitate RT-PCR

Frozen sample of grafts were the same with microarray analysis. 2 ug of total RNA extracted by Trizol (Invitrogen, USA) was used to be reverse-transcripted into cDNA using AMV Reverse Transcriptase Kit (Promega, USA); cDNA, SYBR green PCR Kit and Primers were all added to PCR plate (Axygen Inc, USA). All of the operations are in line with the manufacturer's instructions. Real-time PCR was performance on Applied Biosystems 7500 Real-Time PCR System (California, USA), 2^-△△^Ct^^ was calculated to represent the mRNA expression level of predicted target genes of graft samples. The gene-specific primers used in quantitate RT-PCR were shown in Table 1.

**Table 1:** Primers of 11 predicted target genes for quantitate RT-PCR.

Genes		Primers
IL-6	Forward	TACCACTTCACAAGTCGGAGGC
	Reverse	CTGCAAGTGCATCATCATCGTTGTTC
CaMK II	Forward	AGCCATCCTCACCACTATGCTG
	Reverse	GTGTCTTCGTCCTCAATGGTGG
TAB2	Forward	CATTCAGCATCTCACAGACCCG
	Reverse	CTTTGAAGCCGTTCCATCCTGG
IL-1 beta	Forward	TGGACCTTCCAGGATGAGGACA
	Reverse	GGTCATCTCGGAGCCTGTAGTG
UCP2	Forward	TAAAGGTCCGCTTCCAGGCTCA
	Reverse	ACGGGCAACATTGGGAGAAGTC
RAB9b	Forward	GGAGGTAGATGGACGCTTTGTG
	Reverse	CCACACTGAAGGTTAGCAGGCA
Cyclin M4	Forward	GAGATCCTCGATGAGTCGGACA
	Reverse	GAAGCGATGAGCAGCCAGAAGA
AKT	Forward	GGACTACTTGCACTCCGAGAAG
	Reverse	CATAGTGGCACCGTCCTTGATC
P53	Forward	CCTCAGCATCTTAATCCGAGTGG
	Reverse	TGGATGGTGGTACAGTCAGAGC
TLR3	Forward	GTCTTCTGCACGAACCTGACAG
	Reverse	TGGAGGTTCTCCAGTTGGACCC
RIP140	Forward	AGCCAAGCAGAGTCTCCCATCA
	Reverse	TGCCTTTCGTGAGGTCCATACAG

### Western Blot Analysis

To investigate the expression of putative targets of mmu-miR-152, total protein fractions were purified from allografts and syngrafts as well as normal C3H mice liver. Anti-CaMK II (Cell signaling, USA) and anti-beta-actin (Dawen Biotec, Hangzhou, China) antibody were used for western blot analysis. Equal amounts of protein (40 ug/lane) were resolved by 12% sodium dodecyl sulfate polyacrylamide gel electrophoresis and transferred onto a nitrocellulose membrane. After blocking of non-specific binding sites, membranes were incubated overnight at 4°C with anti-CaMK II antibody (1∶2000), and anti-beta-actin antibody (1∶4000) followed by the corresponding horseradish peroxidase-conjugated secondary antibodies (1∶5000; Dawen Biotec).Then the membranes were developed in the ECL Western detection reagents (Amersham–Pharmacia Biotech, Piscataway, NJ, USA), according to the manufacturer's protocol. The gray value of each band was analyzed and auto-calculated by AlphaView SA software (Alpha Innotech, USA).

### Statistical Analysis

All the data was analyzed using GraphPad Prism 5.0.1 software (GraphPad Prism Software Inc., San Diego, CA) and presented as Mean ± SEM. Student's t test was applied to assess data between different groups and P<0.05 was considered statistically significant.

## Results

### Microarray analysis of differentially expressed miRNAs in allo-/syngeneic grafts

**Figure 1:**
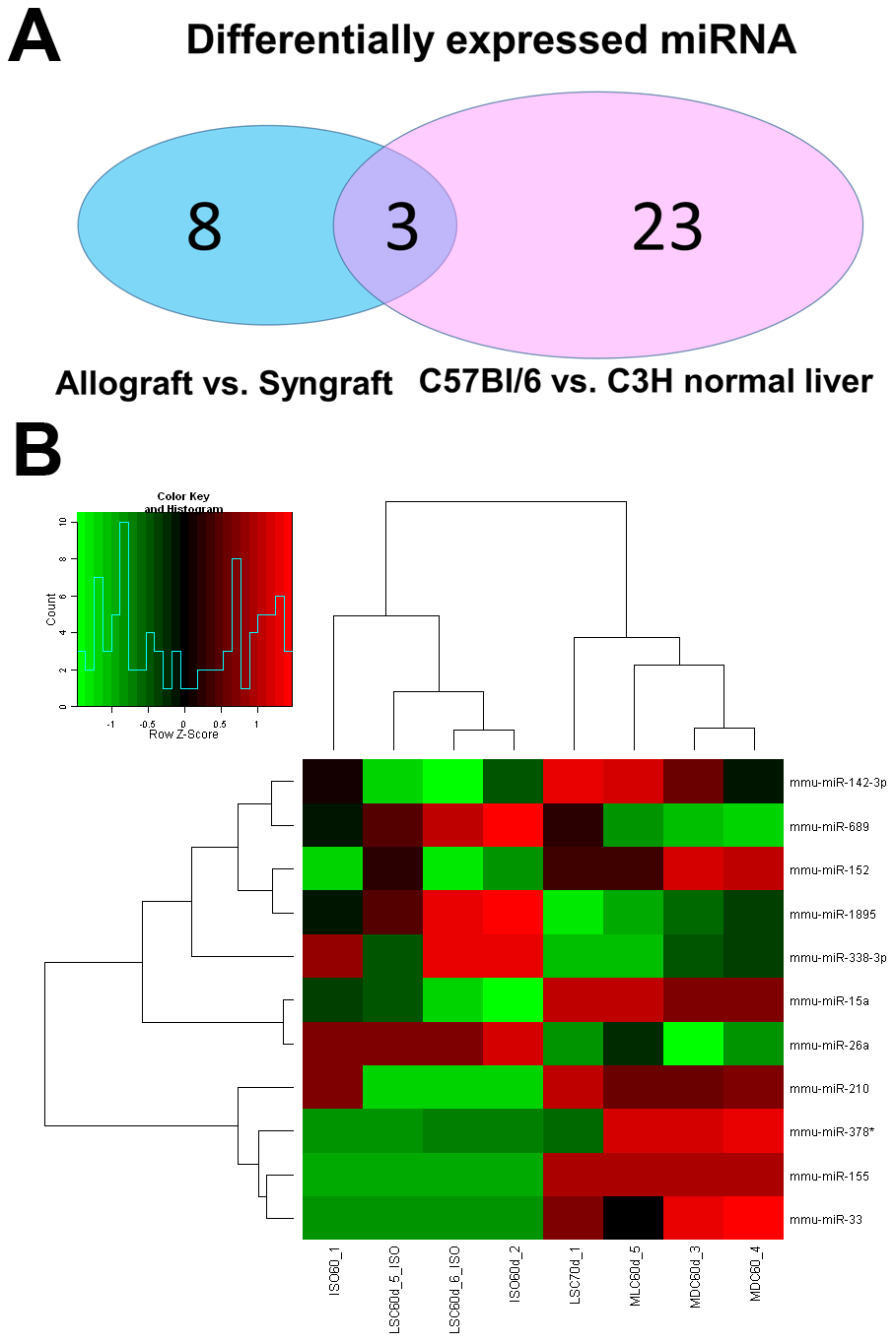
Expression profiles of miRNAs in allo-/syngeneic liver grafts. A: Comparison of observed miRNA in allografts vs. syngrafts and normal C57BL/6 vs. C3H mouse liver. B: miRNA expression profile between allografts and syngrafts. Clustering of the microarray showed the statistically significant (*p<0.05) miRNAs. The columns and rows represent samples and particular miRNAs.

650 mature miRNAs were accessed by the miRNA Chips V12.0 expression profiles. Different miRNAs expressed in allografts/syngrafts, normal liver of C57BL/6 and C3H mice were shown in Figure 1A. 11 different miRNAs from liver transplantation grafts were identified (Table 2), while 26 miRNAs were expressed differently in normal C57BL/6 and C3H mouse liver (Table 3). Among them, 3 miRNAs were both differentially expressed in grafts and normal livers, which meant the other 8 identified miRNAs might be specifically expressed in allografts after liver transplantation.

**Table 2:** Differentially expressed miRNAs in allografts vs. syngrafts.

Different miRNAs in allografts vs. syngrafts		
Systematic Name	P values	Fold change
mmu-miR-142-3p	0.039284	2.096914
mmu-miR-152	0.015278	1.152798
mmu-miR-155	1.25E-09	33.95233
mmu-miR-15a	0.007636	1.427048
mmu-miR-1895	0.018199	0.582312
mmu-miR-210	0.033868	6.880669
mmu-miR-26a	0.009468	0.876999
mmu-miR-33	0.009303	20.795
mmu-miR-338-3p	0.02321	0.363475
mmu-miR-378	0.038569	22.05339
mmu-miR-689	0.03978	0.654757

**Table 3:** Differentially expressed miRNAs in normal C57BL/6 vs. C3H mouse livers.

Different miRNAs in normal liver of B6 vs. C3H	
Systematic Name	Log2 Fold change
mmu-let-7e	1.0795035
mmu-miR-1187	4.6175737
mmu-miR-1198	4.7325196
mmu-miR-125a-5p	8.1163
mmu-miR-142-5p	7.888199
mmu-miR-1895*	−1.3780842
mmu-miR-200b	6.960029
mmu-miR-210*	4.649441
mmu-miR-290-3p	−7.272381
mmu-miR-296-5p	−2.1873841
mmu-miR-29b	4.3295603
mmu-miR-29c	4.5762167
mmu-miR-301a	5.3840694
mmu-miR-30c-2	4.6977553
mmu-miR-31	−7.4403687
mmu-miR-33*	4.537604
mmu-miR-340-5p	7.2943954
mmu-miR-342-3p	8.638982
mmu-miR-362-3p	−1.4627097
mmu-miR-423-5p	4.777275
mmu-miR-425	6.8487062
mmu-miR-455	−7.3192835
mmu-miR-702	−6.9172764
mmu-miR-712	−2.3940206
mmu-miR-802	8.667955
mmu-miR-99b	5.4394116

* MiRNAs differentially expressed both in allografts vs. syngrafts and C57BL/6 vs. C3H normal livers.

Different miRNAs identified by microarray chips with P values and/or Fold-change were listed separately in Table 2. Fold-change>1 means miRNA was up-regulated, while fold-change<1 indicates a downregulated miRNA. A dendrogram of a hierarchical clustering analysis of differentially expressed miRNAs between allografts/syngrafts was shown in Figure 1B.

### Biological Function Analysis of Identified miRNAs between Allografts and Syngrafts

Table 4 showed the main biological functions of the 11 differentially expressed miRNAs between allografts and syngrafts. Some miRNAs had important biological functions in transplantation immunology. For example, miR-142-3p could regulate cytokine-cytokine receptor interaction and Fc gamma R mediated phagocytosis, while miR-152 played a role in endocytosis and membrane associated granulate kinase. Regulation of lymphoid development, apoptosis, Toll-like receptor signaling pathway and nucleotide excision repair was modified by miR-15 and miR-155. The mTOR signaling pathway was correlated with miR-26b, while miR-378 mediated natural killer cell cytotoxicity and TCR signaling pathway. These miRNAs were all related with pro- and anti-apoptotic proteins.

**Table 4:** Main functions of the differentially expressed miRNAs in allografts/syngrafts.

miRNA	Biological Function
miR-142-3p	Cytokine-cytokine receptor interaction,Jak-STAT signaling pathway,Adherens junction,Fc gamma R-mediated phagocytosis,Regulation of actin cytoskeleton,Pathogenic Escherichia coli infection,Focal adhesion,ECM-receptor interaction,Cell adhesion molecules,Regulation of actin cytoskeleton
miR-152	Glycerophospholipid metabolism,Phosphatidylinositol signaling system,RNA degradation,ABC transporters,ABC transporters,Endocytosis,membrane associated guanylate kinase,Neuroactive ligand-receptor interaction
miR-15	Apoptosis,Neuroactive ligand-receptor interaction,muscle contraction,Aldosterone-regulated sodium reabsorption,Toll-like receptor signaling pathway,RIG-I-like receptor signaling pathway,Nucleotide excision repair,Melanogenesis,Lysine degradation,Tight junction,Cardiac muscle contraction,Focal adhesion
miR-155	MAPK signaling pathway,Toll-like receptor signaling pathway,NOD-like receptor signaling pathway
miR-210	Oxidative phosphorylation,O-Glycan biosynthesis,Axon guidance,Glycerophospholipid metabolism
miR-26b	Cytokine-cytokine receptor interaction,Focal adhesion,Renal cell carcinoma,Melanoma,Lysine degradation,Oocyte meiosis,mTOR signaling pathway,Long-term potentiation,Progesterone-mediated oocyte maturation
miR-33	ABC transporters,Lysine degradation,Cell cycle,Cytokine-cytokine receptor interaction,Endocytosis,Focal adhesion,Gap junction,Regulation of actin cytoskeleton,Pathways in cancer,Colorectal cancer,Glioma,Prostate cancer,Melanoma
miR-338-3p	SNARE interactions in vesicular transport
miR-378	Axon guidance,ErbB signaling pathway,Dorso-ventral axis formation,Focal adhesion,Gap junction,Natural killer cell mediated cytotoxicity,T cell receptor signaling pathway,Fc epsrilon RI signaling pathway

### Target Gene Prediction of Differentially Expressed miRNAs

Target gene prediction of all the differentially expressed miRNAs between grafts were performed using the online software and databases. In total, there were 185 target genes predicted for the 11 identified miRNAs (Table 5). MiR-689 was not matched in the database of TargetscanMouse 6.0. Then, we consulted abundant references and performed plenty of information retrieval work. Among all the 185 predicted target genes, 11 of them had an intense relationship with immune regulation of transplantation and were listed in Table 6 with their target protein, gene ID in NICB, along with their correspondent miRNA.

**Table 5:** 185 predicted target genes of the 11 differentially expressed miRNAs in allografts/syngrafts.

Mature miRNA Name	Predicted target protein
mmu-miR-142-3p	FMN1,FYCO1,PRLR,RAB2A,WASL,EGFL6,NPSR1,GORAB,DBX2,BC016423,ITGA8,ESYT3,2210010C04RIK,ZFP943,DCAF12L1,RNF219,RAB12,CTSM,FAM114A1,IL6
mmu-miR-152	OSBPL11,DCP2,MEOX2,BCL2L11,PHACTR2,DCUN1D3,SGCB,ABCA1,EIF2C4,PLAA,ARL6IP1,EPS15,USP32,SGMS1,ARPP19,MAGI1,CDS1,B4GALT5,ABCB7,UCP3,ATP8A1,MNT,INO80,MED12L,ARRDC3,ARHGAP21,S1PR1,QK,TMEM9B,FBN1,SIK1,CAMK IIΑ
mmu-miR-155	RUFY2,GPD1L,TSHZ3,ODZ3,TAB2,IL1BETA
mmu-miR-15a	ZBTB34,CPEB2,RAB9B,ATP7A,ACTR2,PAPPA,C20ORF46,PRKAR2A,DCAF7,EPT1,ZNRF2,PPPDE2,GRID1,ASH1L,ZDHHC22,HTR4,KL,PWWP2B,MYT1L,KCNK10,TRAF3,CCNE1,PDK3,TLK1,ARL2,CAPZA2,COL12A1,LRP6,RASGEF1B,SPTBN2,FGF2,NUP210,SLC20A2,ATP1B4,MGAT4A,ZMYM2,GPATCH8,ITGA2,FZD10,N4BP1,CACNB1,FBXO21,DCLK1,PHF19,LUZP1,KIF23,FAM59A,WNT3A,RS1,ZYX,CLSPN,ABTB2,ODZ2,PLEKHA5,EIF3A,RAD23B,UNCOUPLING PROTEIN-2
mmu-miR-1895	CNNM4,SUPT16H,FBXL16,FAM53C,MNT,NTM,ESPN
mmu-miR-210	GPD1L,C6ORF136,B4GALT5,ELFN2,SYNGAP1,ISCU,KCMF1,NEUROD2,EFNA3,NDUFA4,BDNF,MLL2,AKT,P53
mmu-miR-26a	FAM98A,ZDHHC20,STRADB,HGF,RPS6KA6,LARP1,KIAA1737,TNRC6B,PLOD2,NAP1L5,CHFR,NAB1,ST6GAL2,DNAJC24,SLC25A16,LLPH,SLC25A36,TLR3
mmu-miR-33	ABCA1,SLC12A5,HMGA2,ZNF281,DCUN1D5,PDGFRA,CDK6,SLC25A25,SETD7,RIP140
mmu-miR-338-3p	SETD7,SNAP29,SH2D4A,MTUS1,PRRC2C,TRIM33,ZNF238,ARHGEF10L,HERPUD2
mmu-miR-378	EFNA5,KCND1,DYRK1A,KCNIP2,ARRDC2,QSER1,GRB2,TEX261,GRSF1,PURB,TFCP2L1,SRSF3,GOLT1A

**Table 6:** List for the 11 predicted target genes which had an intense relationship with transplant immune regulation.

miRNA Name	Target Gene	Predicted Protein
mmu-miR-142-3p	NC_000007.13	IL-6
mmu-miR-152	NC_000084.5	CaMK II
mmu-miR-155	NC_000076.5	TAB2
	NC_000068.6	IL-1 beta
mmu-miR-15a	NC_000073.5	UCP2
	NC_000086.6	RAB9b
mmu-miR-1895	NC_000067.5	Cyclin M4
mmu-miR-210	NC_000078.5	AKT
	NC_000077.5	P53
mmu-miR-26a	NC_000074.5	TLR3
mmu-miR-33	NC_000082.5	RIP140

### GO and KEGG Pathway Analyses of the Predicted Target Genes

**Figure 2:**
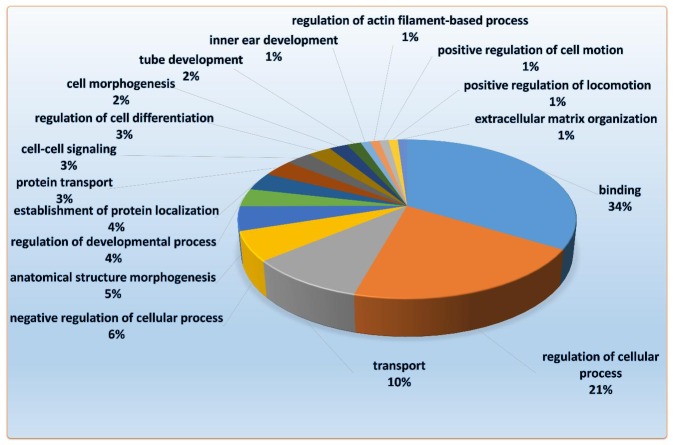
Pie charts showing the distribution of GO categories for the 185 predicted target genes of differentially expressed miRNAs in allografts compared with syngrafts.

In order to have a better understanding of biological function of these identified miRNAs, we performed GO and KEGG pathway analyses for all the predicted target genes. By using Functional Annotation Tool of “Database for Annotation, Visualization and Integrated Discover”, biological functions and effects of these identified miRNAs and their target genes were annotated. The top 19 annotation clusters among the predicted target genes of these differentially expressed miRNAs were shown in Figure 2. GO analyses revealed that 34% of these predicted target genes had important biological functions in binding (protein-protein binding, DNA-protein binding), 21% of them regulated cellular process including DNA binding, transcription regulation and protein synthesis, 10% mainly were involved in cation transport, metal ion transport and protein transport.

As was shown in Table 7, KEGG pathway analysis annotated pathways of all the predicted target genes. Mmu-miR-142-3p was involved in cytokine-cytokine receptor interaction, JAK-STAT signaling pathway, and chemokine signaling pathway, Fc gamma R-mediated phagocytosis and cell adhesion molecules. Mmu-miR-152 could modify RNA degradation, endocytosis, sphingolipid metabolism and phosphatidylinositol signaling system. Mmu-miR-15 family was associated with apoptosis, lysine degradation, calcium signaling pathway, Toll-like receptor signaling pathway, p53 signaling pathway and multiple pathways related with melanoma, prostate cancer, small cell lung cancer, colorectal cancer, basal cell carcinoma. Mmu-miR-155, mmu-miR-26 and mmu-miR-33 played important roles in immune reaction and response (involved in Toll-like receptor signaling pathway) and signal pathway induction (involved in MAPK signaling pathway, mTOR signaling pathway, calcium signaling pathway). Among the targets for differentially expressed miRNAs, mmu-miR-378 had important roles in immune function (involved in natural killer cell mediated cytotoxicity, T cell receptor and B cell receptor signaling pathway in miR-497), signal pathway induction (involved in the calcium signaling pathway, ErbB signaling pathway, MAPK signaling pahtway), and nutrition metabolism (involved in GnRH signaling pathway and the insulin signaling pathway).

**Table 7:** KEGG pathway analysis of the targets of the differentially expressed miRNAs in allografts/syngrafts.

miRNA	KEGG Pathways	Target Protein
mmu-miR-142-3p	Cytokine-cytokine receptor interaction	PRLR
	Chemokine signaling pathway	WASL
	Focal adhesion	ITGA8
	Cell adhesion molecules	-
	Jak-STAT signaling pathway	-
	Fc gamma R-mediated phagocytosis	-
mmu-miR-15	Nucleotide excision repair	RAD23B
	Wnt signaling pathway	WNT3A,NPSR1,HTR4,SGCB,EFNA3,FZD10
	N-Glycan biosynthesis	ST6GAL2,B4GALT5,ZDHHC22,ZDHHC20,MGAT4A
	Focal adhesion	ITGA2
	MAPK signaling pathway	FGF2
	Lysine degradation	MLL2,SETD7,ASH1L
	Calcium signaling pathway	NPSR1,FZD10,SGCB,SLC20A2,HTR4
mmu-miR-152	RNA degradation	DCP2
	ABC transporters	ABCA1,SLC25A16,SLC25A36,ABCB7
	Endocytosis	EPS15
	Sphingolipid metabolism	ST6GAL2,SGMS1
	Tight junction	MAGI1
	Glycerophospholipid metabolism	EPT1,CDS1
	Neuroactive ligand-receptor interaction	S1PR1
	Phosphatidylinositol signaling system	-
mmu-miR-155	MAPK signaling pathway	RNF219,ZDHHC20,GPATCH8,FYCO1,TAB2
	Toll-like receptor signaling pathway	-
	NOD-like receptor signaling pathway	-
mmu-miR-210	Glycerophospholipid metabolism	GPD1L
	O-Glycan biosynthesis	MGAT4A,ST6GAL2,ZDHHC22,ZDHHC20,EPT1,GOLT1A,NPSR1,B4GALT5
	Axon guidance	EFNA5,NTM,FZD10,EFNA3
	Oxidative phosphorylation	NDUFA4
	MAPK signaling pathway	BDNF
mmu-miR-26	Toll-like receptor signaling pathway	TLR3
	Cytokine-cytokine receptor interaction	HGF
	MAPK signaling pathway	SIK1,TLK1,DYRK1A,DCLK1,PKD3,CDK6,STRADB,RPS6KA6
	Lysine degradation	PLOD2
mmu-miR-33	ABC transporters	ABCA1
	MAPK signaling pathway	PDGFRA
	Cell cycle	TLK1,DYRK1A,SIK1,RPS6KA6,CDK6
	Lysine degradation	ASH1L,SETD7
mmu-miR-338-3p	SNARE interactions in vesicular transport	SNAP29
mmu-miR-378	Axon guidance	EFNA3,NTM,EFFNA5
	MAPK signaling pathway	GRB2
	ErbB signaling pathway	-
	Natural killer cell mediated cytotoxicity	-
	T cell receptor signaling pathway	-
	B cell receptor signaling pathway	-
	Insulin signaling pathway	-
	GnRH signaling pathway	-

### Quantitate RT-PCR Analysis of Potential Target Genes

**Figure 3:**
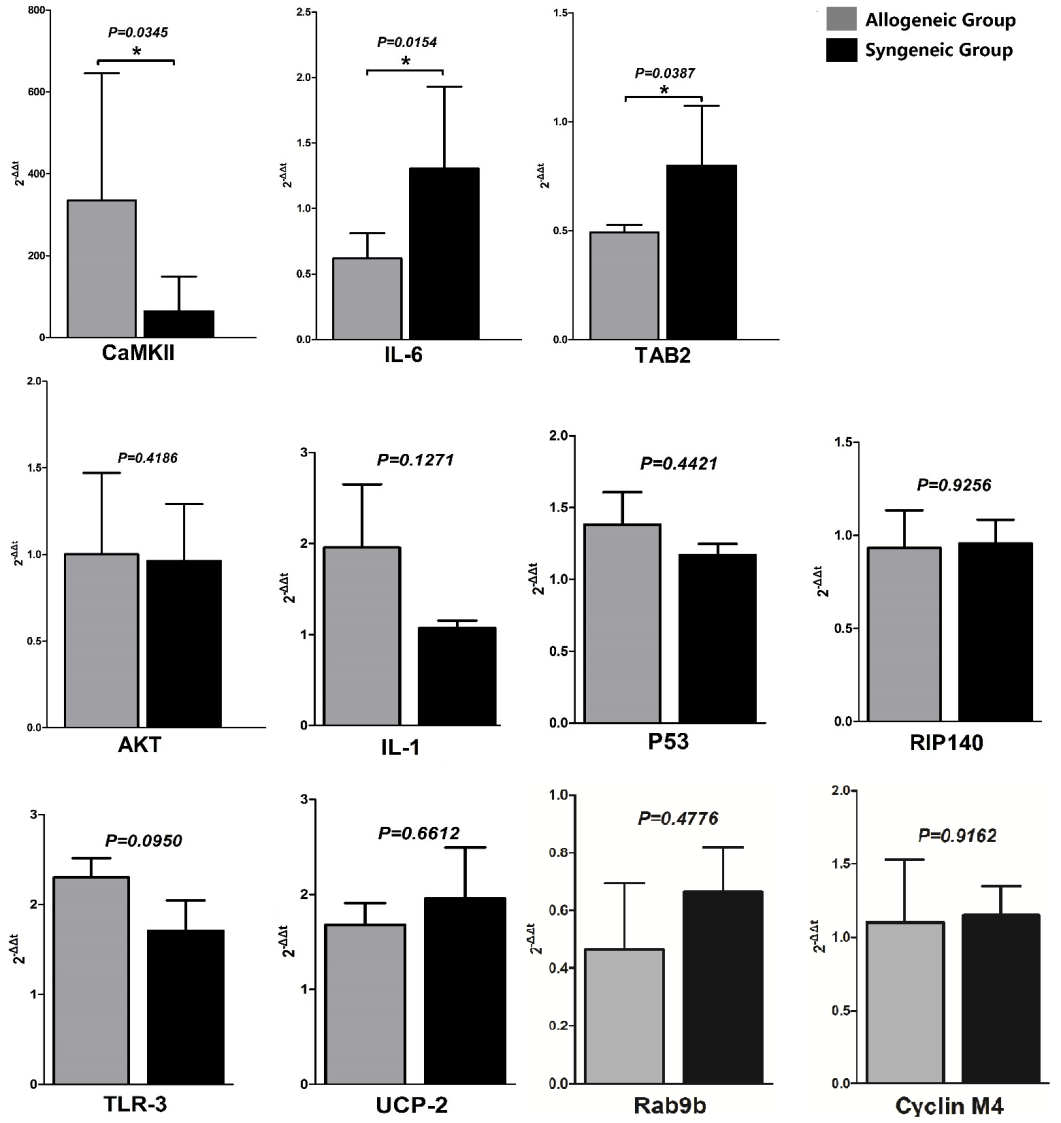
mRNA expression levels of 11 target genes regulated by identified miRNAs. Among them, mRNAs of CaMK II, IL-6 and TAB2 were significantly changed in allografts compared with syngrafts, whose p values was 0.0345, 0.0154 and 0.0387, respectively. mRNAs of IL-6 and TAB2 was down regulated by increased mmu-miR-142-3p and mmu-miR-15, while mmu-miR-152 and mRNA of CaMK II was both increased in allografts compared with syngrafts.

We validated the expression of the identified miRNA correlated well with those of the microarray by quantitate RT-PCR using the same RNA sample for microarray chips. Afterwards, we selected the 11 predicted genes which might have a potential effect on immune tolerance of liver graft and assayed their mRNA level to verify whether the target genes were also significantly changed or not. To our surprise, there were only 3 predicted target genes had a significant change in mRNA level (CaMK II, IL-6, TAB2), while the others were not (AKT, IL-1, P53, RIP140, TLR3, UCP-2, RAB9b, CYCLIN M4), as was shown in Figure 3. The mRNAs levels of IL-6 and TAB2 were down-regulated by increased mmu-miR-142-3p and mmu-miR-155 in allogeneic grafts compared with syngeneic grafts. While highly expressed mmu-miR-152 didn't degrade mRNA of CaMK II. Since the mRNA level of CaMK II was higher in allografts, to verify the exact relationship of mmu-miR-152 and CaMK II, we applied western blot to verify the exact relationship of mmu-miR-152 and CaMK II.

### Western Blot Analysis of CaMK II Expression

**Figure 4:**
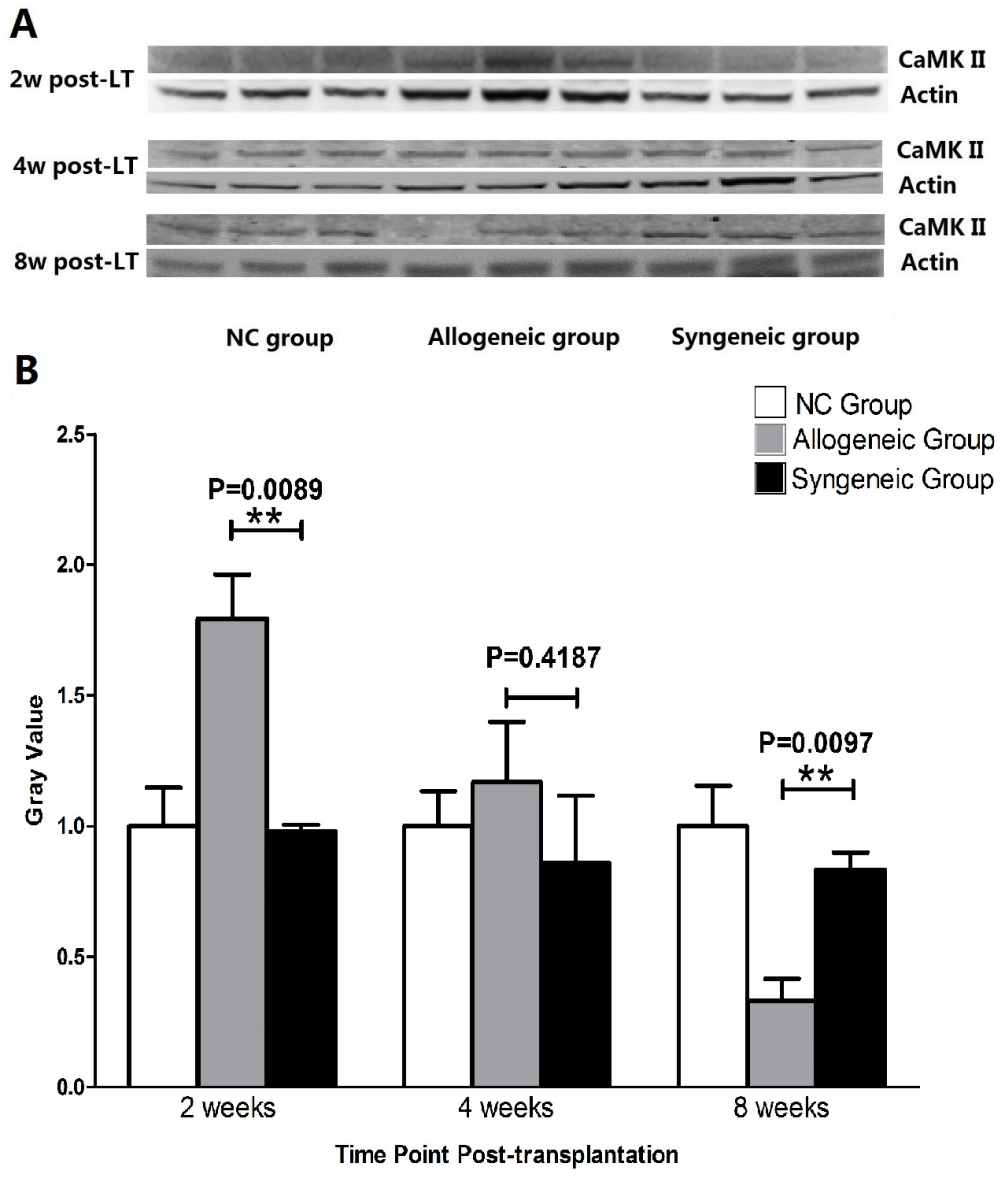
Western blot results showed CaMK II was decreased generally from 2 weeks to 8 weeks post-transplantation. A. Western blot bands of CaMK II and actin in normal livers, allografts and syngrafts at different time point post-transplantation. B. Gray value analysis of bands for CaMK II expression.

The protein expression of CaMK II in allogeneic grafts was down-regulated significantly compared with syngeneic grafts 8 weeks post-transplantation. The gray-values of normal C3H livers, allografts and syngrafts was 1.000+0.1548, 0.3309+0.08488 and 0.8330+0.06698, respectively. Our previous study showed that the expression of CaMK II in allografts was significantly higher than syngrafts 2 weeks after transplantation, with obvious inflammation intra liver grafts. While at 4 weeks post-transplant, there was no difference in expression of CaMK II between allogeneic and syngeneic grafts, as was shown in Figure 4.

## Discussion

Since the phenomenon of “spontaneous immune tolerance” without immunosuppressant was firstly reported by Qian et al, mouse liver transplantation has always been regarded as the ideal model in tolerance research [17], [18]. Li W. et al revealed the increased Foxp3(+)CD25(+)CD4(+) T cells in liver grafts and recipient spleens, along with enhanced CTLA4 expression, was critical for murine liver transplant tolerance induction [19], [20]. However, Steger et al. provided evidence that Tregs was more likely the result of sustained exposure to donor allo-antigens in vivo [21]. Other studies indicated liver sinusoidal endothelial cells were capable of regulating a polyclonal population of T cells with direct allo-specificity through the Fas/Fas ligand pathway [22], [23]. Meanwhile, the underlying mechanisms of spontaneous tolerance still remain to be clarified. To date, no studies have ever reported the expression profile changes and biological functions of miRNAs in tolerated mouse liver grafts.

Our previous study of murine liver transplantation model revealed lymphocytes began to infiltrate into liver grafts during the first week post-operation, the number of infiltrating cells would reach peak in 14 days, and decreased gradually afterwards, immune tolerance would be reached 2 months post-transplantation. Thus, to investigate possible mechanisms involved in tolerated mouse liver graft, allografts and syngrafts 2 months post-transplantation were chosen as our study objects.

By using high-throughput technology of microarray chips, we successfully identified 11 miRNAs which changed in tolerated liver grafts. The profiles of normal C57BL/6 vs. C3H were used to exclude miRNA changes caused by species discrepancy. It means that among the 11 identified miRNAs, mmu-miR-1895, mmu-miR-210 and mmu-miR-33 could be caused by species discrepancy. However, whether they are involved in immune tolerance of mouse liver transplantation requires further investigation. GO and KEGG analysis indicated the potential functions of these identified miRNAs were mainly involved in cytokine-cytokine receptor interaction, immune response, metabolism, and cell cycle and differentiation. Among them, miR-142-3p was involved in Fc gamma R-mediated phagocytosis. IL-6 was predicted to be targeted by miR-142-3p. It may play a critical and specific role in regulating IL-6 production by the dendritic cells (DCs) after LPS stimulation [24]. In vivo delivery of a miR-142-3p regulated transgene could induce antigen-specific regulatory T cells and promoted immunologic tolerance [25], [26]. MiR-155 was reported to have a relationship with inflammation and CD56^+^, CD14^+^ monocytes or the development of DCs. It can promote the secretion of pro-inflammatory cytokines, such as IL-1, IL-2, IL-6, IFN, CXCL1 and CXCL9 [27]–[29]. MiR-15 family was reported to be involved in cell cycle, apoptosis and nucleotide excision repair. It could regulate multiple cellular pathways such as calcium signaling, Toll-like receptor signaling pathway, p53 signaling pathway, Wnt signaling pathway and MAPK signaling pathway [30], [31]. While miR-33, which has been reported to have a protective effect on macrophage induced in inflammation, could regulate the processes of endocytosis, gap junction, focal adhesion, lysine degradation and cytokine-cytokine receptor interaction [32], [33]. MiR-152 was involved in phosphatidylinositol signaling system, and could regulate gene of CaMK II. It was reported that mmu-miR-152 combined with CaMK II alpha to inhibit innate immune response induced by DCs, and down-regulated the synthesis of IL-12, IL-6, TNF-alpha and IFN-beta to impede the maturity of DCs and activation of T cells [34]-[36]. MiR-378 was reported to be involved in regeneration of stem cells and KEGG analysis revealed it may play an important role in natural killer cells mediated cytotoxicity, T cell, B cell receptor signaling pathways [37]-[39].

After validation of the identified miRNAs, we chose 11 target genes mostly related with immune regulation to verify whether the mRNAs were changed. The mRNA levels of IL-6, TAB2 were down regulated and mRNA of CaMK II was up regulated in the tolerated allogeneic liver grafts. Since miR-142-3p (target protein IL-6), miR-155(target protein TAB2) and miR-152 (target protein CaMK II) were increased in allografts, we could speculate that miR-142-3p and miR-155 may combine with mRNAs of IL-6 and TAB2 and induce their degradation. TAB2 was proved to be essential for B cell activation leading to Ag-specific Ab responses, as well as B1 and marginal zone B cell development [40], Xu and his colleagues had already provided evidence that miR-155 regulates immune modulatory properties by targeting TAB 2 [41]. On the other hand, IL-6 have been studied for decades of years. It was proved to be pro-inflammatory cytokine that can be observed in many pathogenesis conditions such as systemic sclerosis, ankylosing spondylitis as well as in organ or cell transplantation [42]-[44]. While other studies revealed that IL-6 was correlated with immunosuppressive effects of ESCs and MSCs. What's more, IL-6 gene expression changes were involved in eight different immune response pathway [45]. The role of IL-6 in liver tolerance might be non-specific.

Thus, we focused our study on miR-152 and CaMK II. To clarify the relationship of miR-152 and mRNA of CaMK II, we displayed western bolt to check expression of CaMK II at protein level. As shown in Figure 4, protein of CaMK II was down regulated compared with syngeneic liver graft, thus, we could presume that mediated by the sequence complementarity, miR-152 bind to 3′-UTR of mRNA of CaMK II to inhibit the initiation of protein translation by connecting the cap of mRNA or lead to post-initiation inhibition by nascent protein degradation and ribosome drop-off [46], [47]. In this potential manner, miR-152 silenced the translation of CaMK II instead of degrading its mRNA. Our previous study showed that expression of CaMK II was down regulated gradually post-transplantation (Figure 4). Two months later, the expression of CaMK II was even lower than normal C3H mouse?Our study were anastomotic with Liu's results and confirmed a critical role of mmu-miR-152, which combined with CaMK II to induce immune tolerance in liver grafts of murine model.

Our study firstly revealed the expression changes of miRNAs in tolerated mouse liver graft and their biological functions. IL-6, TAB2 and CaMK II may have important effect in tolerance induction. Especially the change of CaMK II was accordant with the progress of mouse liver graft from rejection to tolerance post-transplantation.

## Conclusion

In summary, our present study revealed substantially a remarkable miRNA profiles differentially expressed in tolerated liver grafts and their biological functions. GO and KEGG analyses indicated their predicted target gene may be involved in cell cycle, nutritional metabolism, immune response and signal induction. Validated by quantitate RT-PCR and western blot, we proclaimed that increased mmu-miR-142-3p and mmu-miR-155 down regulated mRNA of IL-6 and TAB2, while highly expressed mmu-miR-152 could silence mRNA of CaMK II and down regulate the translation of CaMK II, which may play an important role in tolerance induction, indicating it could be a potential spot for clinic application. But how CaMK II play a part in tolerance induction and how we can use it for clinic treatment still require further investigations.
